# Impact of high prebiotic and probiotic dietary education in the SARS-CoV-2 era: improved cardio-metabolic profile in schizophrenia spectrum disorders

**DOI:** 10.1186/s12888-022-04426-9

**Published:** 2022-12-12

**Authors:** Alfonso Sevillano-Jiménez, Manuel Romero-Saldaña, Juan Antonio García-Mellado, Lorena Carrascal-Laso, María García-Rodríguez, Rafael Molina-Luque, Guillermo Molina-Recio

**Affiliations:** 1 Montilla Community Mental Health Unit. Mental Health Clinical Management Unit. Reina Sofia University Hospital. Avda. Andalucía, nº11, 14550 Montilla (Córdoba), Spain; 2 Department of Nursing, Pharmacology and Physiotherapy, University of Cordoba. Lifestyles, Innovation and Health (GA-16). Maimonides Biomedical Research Institute of Cordoba (IMIBIC), Avd Menéndez Pidal S/N, 14004 Córdoba, Spain; 3 Psychiatry Service, Zamora Provincial Hospital. Zamora Welfare Complex, C/Hernán Cortés, nº 40, 49021 Zamora, Spain; 4 Department of Nursing and Nutrition, Biomedicine Sciences and Health Faculty, European University. C/Tajo S/N, 28670 Villaviciosa de Odón (Madrid), Spain; 5 Lifestyles, Innovation and Health (GA-16), Maimonides Biomedical Research Institute of Cordoba (IMIBIC), Avd Menéndez Pidal S/N, 14004 Córdoba, Spain

**Keywords:** Metabolic Syndrome, Cardiometabolic Risk Factors, Schizophrenia Spectrum and Other Psychotic Disorders, Nursing, SARS-CoV-2

## Abstract

**Background:**

The development of new aetiological premises, such as the microbiota-gut-brain axis theory, evidences the influence of dietary and nutritional patterns on mental health, affecting the patient's quality of life in terms of physical and cardiovascular health. The aim was to determine the impact of a nutritional programme focused on increasing the intake of prebiotic and probiotic food on cardio-metabolic status in individuals with schizophrenia spectrum disorders in the contextual setting of the SARS-CoV-2 era.

**Methods:**

A randomised clinical trial (two-arm, double-blind, balanced-block, six-month intervention) was conducted in a group of 50 individuals diagnosed with schizophrenia spectrum disorder during the SARS-CoV-2 confinement period. The control group received conventional dietary counselling on an individual basis. In the intervention group, an individual nutritional education programme with a high content of prebiotics and probiotics (dairy and fermented foods, green leafy vegetables, high-fibre fruit, whole grains, etc.) was established. Data on cardiovascular status were collected at baseline, three and six months. In addition, anthropometric parameters were analysed monthly.

**Results:**

Forty-four subjects completed follow-up and were analysed. Statistical differences (*p* < 0.05) were found in all anthropometric variables at baseline and six months of intervention. A 27.4% reduction in the prevalence of metabolic syndrome risk factors in all its components was evidenced, leading to a clinically significant improvement (decrease in cardiovascular risk) in the intervention group at six months.

**Conclusions:**

The development of a nutritional programme focused on increasing the dietary content of prebiotics and probiotics effectively improves the cardio-metabolic profile in schizophrenia spectrum disorders. Therefore, nursing assumes an essential role in the effectiveness of dietary interventions through nutritional education and the promotion of healthy lifestyles. Likewise, nursing acquires a relevant role in interdisciplinary coordination in confinement contexts.

**Trial registration:**

The study protocol complied with the Declaration of Helsinki for medical studies; the study received ethical approval from referral Research Ethics Committee in November 2019 (reg. no. 468) and retrospectively registered in clinicaltrials.gov (NCT04366401. First Submitted: 28th April 2020; First Registration: 25th June 2020).

**Supplementary Information:**

The online version contains supplementary material available at 10.1186/s12888-022-04426-9.

## Introduction

Undoubtedly, the traditional therapeutic approach in psychiatry has perceived the role of nutrition as a minor intervention, especially in severe and long-term mental disorders (LTMD) such as schizophrenia [1]. However, advances in the last decade in terms of Nutritional Psychiatry (focused on the impact of eating patterns on how people feel emotionally) and the presence of new unhealthy dietary practices have contributed to understanding the role of nutritional habits on the central nervous system (CNS) functioning and possible mechanisms or aetiological pathways of psychiatric disorders [1–3].

Similarly, with the development of holobiont theory and the evolution of metagenomics, the concept of the "Microbiota-Gut-Brain Axis " [2, 3] emerged and is currently the subject of study in mental health as part of Nutritional Psychiatry. This term refers to the bidirectional communication pathway between the CNS, gastrointestinal tract, and microbiota (MI) [2, 4], which determines the organism's normal functioning: development and maturation of the CNS, metabolism, immune response, and systemic inflammation [3–7]. Thus, the existence of possible modifications in the concentration of this biota (determined by dietary patterns) can trigger homeostatic alterations or aggravate pathogenic states; a fact commonly referred to as dysbiosis [1, 3, 6, 7]. Thus, according to the theory of low-grade systemic inflammation, a cascade of pro-inflammatory agents capable of modifying both the integrity and permeability of enterocytes [1, 5, 6, 8] is generated when a state of dysbiosis occurs. These agents trigger the release of pro-inflammatory cytokines (tumour necrosis factor ἀ or interleukins type 6 or 1ß) [4–6], which leads to synergies between inflammation, increased oxidative stress and imbalance in energy homeostasis [8].

The current context of an international health emergency and the measures implemented by governments to deal with the SARS-CoV-29 pandemic (border closures, social distancing and home quarantine) have favoured the deterioration of lifestyles [9–12]. This fact has increased the risk of homeostatic dysregulation in the particularly vulnerable population (LTMD) [9, 11]. In addition, the modification of dietary patterns may have altered the functioning of the microbiota-gut-brain axis.

## Background

Evidence shows a high rate of disability, morbidity and mortality (up to 20% higher) [14, 15] in people suffering from psychiatric disorders, especially in patients with LTMD [1, 2, 13–16]. Moreover, these alterations are closely linked to the development of Metabolic Syndrome (MS) [2, 14, 16, 17], which is considered a determining factor in the patient's physical health and can triple the incidence of cardio-metabolic diseases (diabetes mellitus, ischaemic heart disease, etc.) [8, 18].

The main etiopathogenic determinants of MS in these patients are linked to the characteristics of the disease itself and resistance to optimal physical health and lifestyle care [13–16]. In addition, it is essential to note that the psychopharmacological treatment usually prescribed for these patients directly impacts the cardio-metabolic health of the psychiatric patient [13, 15, 16].

Despite the magnitude and severity of the problem, interventions aimed at modifying lifestyles do not play a prominent role in the routine clinical practice of the psychiatric population [2, 14, 17]. Therefore, it is vital to intervene on these factors (including dietary patterns) to improve cardio-metabolic dysfunction. This type of action should be considered as a complement to the conventional therapeutic approach [2–4, 19].

In this regard, some dietary interventions effectively modulate the gut microbiota through symbiotic products. These are a range of nutritional products and food supplements that include probiotics and prebiotics that have a health benefit for the host [20]. In this regard, the use of "psychobiotics" [7, 21–24], a term that refers to the set of probiotic and/or prebiotic substances whose administration has health benefits for psychiatric patients, is noteworthy [22, 24]. Probiotics include micro-organisms from the intestinal biota that, when administered in adequate amounts, benefit the host (notably the genera Lactobacillus and Bifidobacterium, among others) [4, 5, 22–24]. On the other hand, prebiotics is non-digestible dietary fibre (fructooligosaccharides and oligosaccharides, inulin or pectins) [1] and promote optimal growth and development of probiotics in the gastrointestinal tract, reducing pathogenic microbiota [3, 4, 24].

In short, the future of new models of care in Mental Health should include the focus and promotion of the approach and management of nutritional factors [25], highlighting the educational tool developed by nurses, which may represent the cornerstone in achieving optimal health outcomes.

Therefore, this study aimed to assess the impact of a high-symbiotic diet on metabolic and cardiovascular health outcomes in patients diagnosed with a schizophrenia spectrum disorder in confinement and social restriction due to the SARS-CoV-2 pandemic.

## Materials and methods

### Study design

**Fig. 1:**
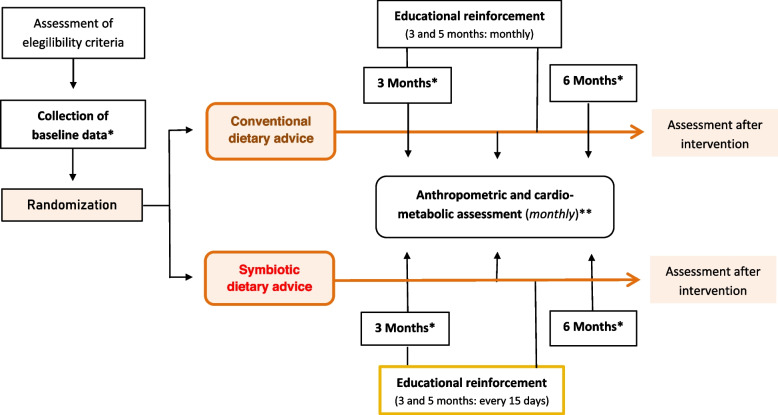
Study Design. *Data collected at baseline, 3 and 6 months of intervention: (1) Biochemical profile. ** Data collected at baseline and monthly during intervention: (1) Anthropometric data (weight, height, Body Mass Index -BMI-, waist circumference and waist-to-height ratio -WHtR-); (2) Cardio-metabolic data (systolic blood pressure, diastolic blood pressure and heart rate)

A controlled, double-blind, two-arm, parallel design, balanced-block, randomised, 6-month intervention clinical trial was developed in psychiatric patients diagnosed with schizophrenia spectrum disorders. The study design is shown in Fig. 1.

### Population

The sample was selected from the referral Psychiatry Service from June 2020 to February 2021. Inclusion criteria were: (1) patients diagnosed on the spectrum of schizophrenia (without distinction by type), according to criteria DSM-5 and/or ICD-11; (2) age between 18–65 years; (3) absence of gastrointestinal comorbidity that contraindicates the use of prebiotics and/or probiotics (intolerance, explosive diarrhoea, acute abdominal pain, etc.); (4) to show clinical stability for six months before the beginning of the study (absence of psychiatric hospitalisation, maintenance of the level of functionality, and lack of social and occupational absenteeism); (5) to manifest agreement to participate in the study and to sign of informed consent.

However, participants were excluded if: (1) suffered from a somatic or neurocognitive situation that prevents participation and collaboration in the fulfilment of the protocol; (2) followed standardised dietary planning not modulated by the population under study (catering, institutional or collective feeding, etc.); (3) refused to participate in the study.

### Sample size

A sample size of 22 individuals was estimated (11 for the control group -CG- and 11 for the intervention group -IG-). A power of 80%, a confidence of 95%, and a risk/prevalence difference of Metabolic Syndrome of 63% post-intervention were also expected [26]. The researchers established the final size of 50 individuals (25 in each group) to minimise the effect of possible losses.

### Intervention

**Fig. 2:**
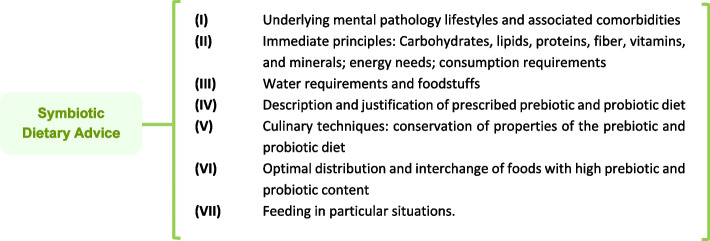
Symbiotic dietary counselling: structure of consultations for the Intervention Group

Advanced practice nurses developed nutritional intervention and education. The CG consisted of those participants who received regular dietary advice (energy needs; immediate principles and consumption requirements -carbohydrates, lipids, proteins, fibre, vitamins, and minerals-; water requirements; regular physical activity) [27] on an individual basis. On the other hand, the intervention group was established individually through intensive nutritional advice [28] with high symbiotic content (Fig. 2). In both intervention groups, specialised nurses used educational resources of visual support during the consultations (Figure S1). Dietary education consisted of increasing the consumption of fermented foods, whole grains, green leafy vegetables and fruits high in dietary fibre, among others. A 6-month individual nutrition education program was implemented (with two months of educational reinforcement, every 15 days for the IG and monthly for the CG). Cardio-metabolic data on a biochemical profile were collected by nursing staff through blood sampling at baseline, 3 and 6 months. Anthropometric variables (BMI, waist-to-height ratio (WHtR), blood pressure, heart rate and waist circumference) were collected monthly by trained nurses, following international protocols [29] (Table S1). The risk of cardiovascular events at ten years was assessed using the REGICOR [30] and SCORE [31] risk functions after six months of intervention. These functions are based on systolic and diastolic blood pressure, age, sex, HDL cholesterol, and smoking, among others.

Finally, to assess adherence, IG participants completed a weekly record of the main dishes/foods consumed with a high prebiotic and probiotic content (fermented foods, whole grains, green leafy vegetables, fruits, etc.), which can be found in the Table S2.

### Data analysis

The quantitative variables have been presented with mean and standard deviation, whereas the qualitative ones with frequencies and percentages. The Kolmogorov–Smirnov test was used for the study of normality in quantitative variables. Student's t-test for paired data, Pearson's correlation coefficient and repeated-means ANOVA, were used to study the relationship between quantitative variables. Chi-square with its corrections (Fisher or Yates) and the McNemar test were computed to study the association between qualitative variables. If the homoscedasticity criterion were not met, non-parametric versions of the previous tests were carried out. For all statistical analyses, a probability of alpha error of less than 5% (*p* < 0.05) and a 95% confidence interval was accepted. SPSS (version 25.0) and EPIDAT (version 4.2) software were used for statistical analysis.

## Results

**Fig. 3:**
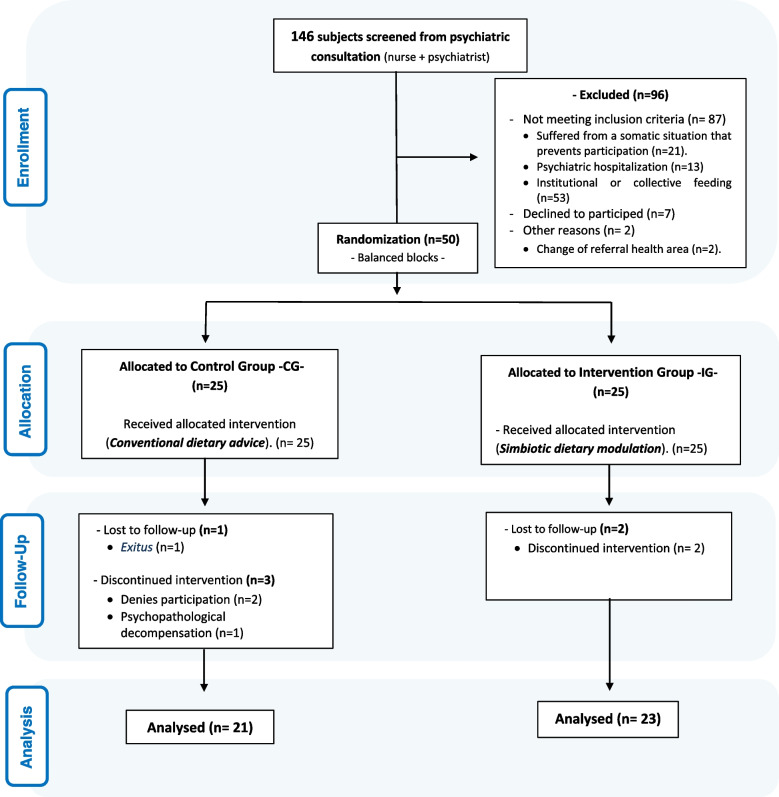
CONSORT flow diagram

During the recruitment period, the eligible population was 50 subjects. Six participants were excluded during the intervention phase. Finally, the study was completed by 21 subjects in the CG and 23 in the IG. The flow chart of the participants is shown in Fig. 3.

32 (72.7%) men and 12 women participated, with a mean age of 49.2 ± 11.9 years. The leading psychiatric diagnosis was schizophrenia (*n* = 37; 84.1%), with a mean duration of illness of 21.6 ± 12.4 years. The mean consumption of intoxicants was 29 smokers (65.9%) for tobacco, followed by 10 subjects who consumed cannabis (22.7%) and 6 participants who reported drinking alcohol (13.6%). Regarding the number of subjects with an associated cardio-metabolic risk factor diagnosis, 14 subjects (31.8%) had dyslipidaemia, 10 (22.7%) hypertension (22.7%), and 7 (15.9%) suffered from diabetes mellitus. Likewise, the prevalence of MS was 43.2% (19 subjects), not including all those with associated cardio-metabolic pathology already diagnosed [20 (45.5%)]. That is, we found a high percentage of subjects with cardiovascular and metabolic alterations prior to the intervention phase.

Finally, the baseline analysis of the dependent variables showed significant differences between the groups analysed for HDL-C, weight, waist circumference and BMI. Tables 1 and 2 contain the baseline characteristics of the independent and dependent variables, respectively, showing homogeneity between both allocation groups.

**Table 1:** Sample characteristics *(independent variables):* Baseline

**Variables**	**TOTAL** (*n* = 44)	**Men** (*n* = 32)	**Women** (*n* = 12)	***P***
***Socio-demographic variables***				
- Age (years)	49.2 (11.9)	50.7 (10.1)	45 (15.5)	0.897
- Legal representative				
**•** No	36 (81.8%)	28 (63.8%)	8 (18.2%)	0.185
**•** Yes	8 (18.2%)	4 (9.1%)	4 (9.1%)	
- Household composition				
**•** Individual	12 (27.3%)	10 (22.8%)	2 (4.5%)	0.285
**•** Horizontal	3 (6.8%)	1 (2.3%)	2 (4.5%)	
**•** Complete	3 (6.8%)	3 (6.8%)	0 (0%)	
**•** Own family home	7 (15.9%)	4 (9.1%)	3 (6.8%)	
**•** Other: Supervised flat	19 (43.2%)	14 (31.8%)	5 (11.4%)	
- Economic level				
**•** High	6 (13.6%)	5 (11.4%)	1 (2.3%)	0.754
**•** Medium	26 (59.1%)	19 (43.2%)	7 (15.9%)	
**•** Low	12 (27.3%)	8 (18.2%)	4 (9.1%)	
- Level of education				
**•** Uneducated	4 (9.1%)	3 (6.8%)	1 (2.3%)	0.009
**•** Primary	19 (43.2%)	9 (20.5%)	10 (22.7%)	
**•** Secondary	17 (38.6%)	16 (36.4%)	1 (2.3%)	
**•** University	4 (9.1%)	4 (9.1%)	0 (0%)	
- Area of residence				
**•** Urban	38 (86.4%)	29 (65.9%)	9 (20.5%)	0.321
**•** Rural	6 (13.6%)	3 (6.8%)	3 (6.8%)	
***Clinical Variables***				
-Psychiatric diagnosis				
**•** Schizophrenia	37 (84.1%)	26 (59.1%)	11 (25%)	0.608
**•** Schizoaffective Disorder	5 (11.4%)	4 (9.1%)	1 (2.3%)	
**•** Delusional Disorder	2 (4.5%)	2 (4.5%)	0 (0%)	
- Duration of illness (years)	21.6 (12.4)	22.4 (11)	16.6 (15.9)	0.715
- Age at first hospitalisation (years)	31.4 (11)	30.7 (10.8)	33.2 (11.5)	0.572
- Consumption of toxics				
**•** No	15 (34.1%)	9 (20.5%)	6 (13.6%)	0.284
**•** Yes	29 (65.9%)	23 (52.3%)	6 (13.6%)	
- Type of toxics				
**•** Alcohol	6 (13.6%)	5 (11.3%)	1 (2.3%)	1.00
**•** Tobacco	29 (65.9%)	23 (52.3%)	6 (13.6%)	
**•** Cocaine	3 (6.8%)	2 (4.5%)	1 (2.3%)	
**•** Opioids	2 (4.5%)	1 (2.3%)	1 (2.3%)	
**•** Amphetamines	3 (6.8%)	2 (4.5%)	1 (2.3%)	
**•** Cannabis	10 (22.7%)	7 (15.9%)	3 (6.8%)	
- Cardio-metabolic diagnosis				
**•** No	24 (54.5%)	15 (34.1%)	9 (20.5%)	0.095
**•** Yes	20 (45.5%)	17 (38.6%)	3 (6.8%)	
- Type Cardio-metabolic diagnosis				
**•** AHT	10 (22.7%)	8 (18.2%)	2 (4.5%)	0.059
**•** DM	7 (15.9%)	5 (11.4%)	2 (4.5%)	
**•** Hyperlipemia	14 (31.8%)	13 (29.6%)	1 (2.2%)	
***Therapeutic Variables***				
- Reason for Change: Antipsychotic Treatment				
**•** Unchanged	31 (70.5%)	20 (45.5%)	11 (25%)	0.302
**•** Lack of efficiency	5 (11.4%)	5 (11.4%)	0 (9.1%)	
**•** Tolerability/safety issues	2 (4.5%)	1 (2.3%)	1 (2.3%)	
**•** Patient's own choice	3 (6.8%)	1 (2.3%)	2 (4.5%)	
**•** Other: Clinical improvement	3 (6.8%)	2 (4.5%)	1 (2.3%)	

*AHT* Arterial hypertension, *DM* diabetes mellitus

**Table 2:** Sample characteristics *(dependent variables):* Baseline

**Variables**	**TOTAL** (*n* = 44)	**Men** (*n* = 32)	**Women** (*n* = 12)	***P***
***Biochemical profile***				
- Glucose *(mg/dL)*	94.1 (13.7)	94.6 (14)	92.7 (13.2)	0.668
- HbA1c *(%)*	5.4 (0.3)	5.5 (0.3)	5.3 (0.3)	0.118
- Cholesterol *(mg/dL)*	186.7 (43.4)	182.3 (47.1)	198.5 (30.2)	0.224
- Triglycerides *(mg/dL)*	131.6 (71)	145.6 (75.7)	94.1 (38.3)	0.026
- LDH *(IU/L)*	185.9 (26.9)	185 (22.7)	94.1 (38.3)	0.948
- C-HDL *(mg/dL)*	49.9 (14.1)	45 (10.8)	63.1 (13.9)	< 0.001
- C-LDL *(mg/dL)*	112.1 (34.7)	110.5 (36.9)	116.3 (28.9)	0.594
- Total cholesterol/C-HDL *(mg/dL)*	3.9 (1.2)	4.1 (1.2)	3.2 (0.8)	0.037
***Anthropometric Profile***				
- Weight *(kg)*	81.4 (17.6)	87.6 (15)	64.6 (12.3)	< 0.001
- Waist circumference *(cm)*	101.9 (17)	104.4 (18.6)	95.2 (9.7)	0.01
- BMI *(kg/m2)*	28.5 (5)	29.6 (5)	64.6 (12.3)	0.011
- WHtR	0.6 (0.1)	0.6 (0.1)	0.6 (0.06)	0.866
- Height (cm)	168.5 (9.2)	172.1 (6.2)	158.7 (9)	0.245
***Cardiovascular Profile***				
- SBP *(mmHg)*	127.2 (15)	128.2 (14.9)	124.5 (15.5)	0.290
- DBP *(mmHg)*	84.2 (10.7)	85.4 (10.6)	80.7 (10.5)	0.204
- HR *(bpm)*	84.8 (14.5)	83.8 (16.5)	87.5 (7.1)	0.186
- Metabolic Syndrome				
No	25 (56.8%)	16 (36.4%)	9 (20.5%)	0.136
Yes	19 (43.2%)	15 (36.4%)	3 (6.8%)	
***Therapeutic Variables***				
- Nº of associated antipsychotic	1.3 (0.5)	1.3 (0.5)	1.3 (0.5)	0.969
- DDD antipsychotics *(mg)*	271.4 (242.5)	284.5 (241.1)	236.4 (253.3)	0.541

*HbA1c* glycosylated haemoglobin, *IFCC* International Federation of Clinical Chemistry and Laboratory Medicine, *LDH* lactate dehydrogenase, *C-HDL* high-density lipoprotein, *C-LDL* low-density lipoprotein, *BMI* body mass index, *WHtR* waist-to-height ratio, *SBP* systolic blood pressure, *DBP* diastolic blood pressure, *HR* heart rate, *Antipsychotic DDD* defined daily dose antipsychotics

Table 3 shows changes in outcome variables at baseline and six months of intervention in CG and IG, respectively. Intragroup analysis showed a significant improvement (*p *< 0.05) in all anthropometric variables in the IG. However, no statistically significant differences were observed in the biochemical and cardiovascular profile and the number of antipsychotics and prescribed dose. Regarding the overall inter-group analysis, no statistically significant results were shown.

**Table 3:** Modifications in Metabolic Syndrome factors: control group and experimental group

	**Control Group** (*n* = 21)			**Intervention Group** (*n* = 23)				
**Variables**	**Basal**	**6 months**	***p***	**Basal**	**6 months**	***p***	P*	P**
***Biochemical profile***								
- Glucose *(mg/dL)*	94.5 (16.8)	102.6 (18.9)	0.03	93.7 (10.4)	97.5 (13.5)	0.023	0.814	0.259
- HbA1c *(%)*	5.4 (0.4)	5.5 (0.3)	0.038	5.4 (0.3)	5.4 (0.6)	0.948	0.887	0.768
- Cholesterol *(mg/dL)*	173.2 (45.4)	171 (43.9)	0.664	199.1 (38.4)	179.4 (64.3)	0.072	0.115	0.155
- Triglycerides *(mg/dL)*	121.1 (67.4)	137.5 (75.8)	0.352	141.1 (74.3)	134.2 (69.4)	0.584	0.235	0.981
- LDH *(IU/L)*	182.5 (22.5)	172.4 (29.9)	0.141	189 (30.6)	172.4 (29.9)	0.049	0.533	0.647
- C-HDL *(mg/dL)*	50.2 (15.4)	48.4 (15.1)	0.067	49.7 (13.1)	52.4 (24.9)	0.611	0.962	0.760
- C-LDL *(mg/dL)*	100.4 (33.8)	96.9 (33.7)	0.446	123 (32.4)	119.3 (41.6)	0.7	0.048	0.067
- Total cholesterol/C-HDL *(mg/dL)*	3.6 (0.9)	3.6 (0.8)	0.522	4.2 (1.3)	4.2 (1.3)	0.905	0.058	0.180
***Anthropometric Profile***								
- Weight *(kg)*	76.6 (18)	75.8 (17.7)	0.382	85.7 (16.3)	81.3 (14.6)	< 0.001	0.086	0.275
- Waist circumference *(cm)*	100.9 (13.3)	101.2 (13.5)	0.818	105.7 (11.5)	102 (11.7)	< 0.001	0.397	0.981
- BMI *(kg/m2)*	27.5 (5.2)	27.2 (5.3)	0.323	29.5 (4.8)	27.9 (4.3)	< 0.001	0.307	0.869
- WHtR	0.59 (0.12)	0.61 (0.07)	0.345	0.61 (0.07)	0.59 (0.06)	< 0.001	0.597	0.378
***Cardiovascular Profile***								
- SBP *(mmHg)*	125.5 (16.3)	129.8 (11.2)	0.124	128.7(13.9)	126.8 (10.6)	0.593	0.391	0.359
- DBP *(mmHg)*	85.5 (9.7)	82.3 (7.9)	0.846	85.6 (11.5)	80.8 (7.5)	0.039	0.548	0.814
- HR (bpm)	88.5 (16.4)	87.4 (14.2)	0.757	81.4 (12)	80.8 (9.4)	0.792	0.110	0.226

*p*: intragroup statistical significance; p*: baseline intergroup statistical significance; p**: 6 months intergroup statistical significance; *HbA1c *Glycosylated haemoglobin, *LDH *Lactate dehydrogenase, *C-HDL *High-density lipoprotein, *C-LDL *Low-density lipoprotein, *BMI *Body mass index, *WHtR *Waist-to-height ratio, *SBP *Systolic blood pressure, *DBP *Diastolic blood pressure, *HR *Heart rate, *CG Evol*. Control group evolution, *IG Evol *Intervention group evolution, *% Balance * Percentage variation, *REGICOR *Calculation of 10-year coronary heart disease risk, *SCORE *Systematic coronary risk assessment for Spain, Antipsychotic *DDD *Defined daily dose antipsychotics

**Fig. 4:**
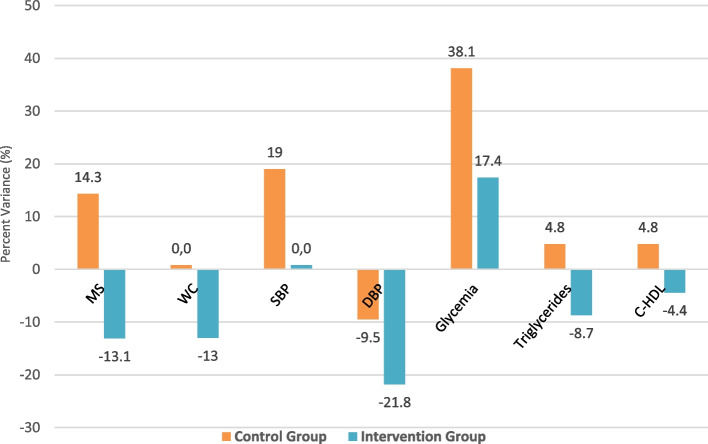
Evolution in allocation groups at 6 months: Metabolic Syndrome and Lipid Profile. MS: metabolic Syndrome (*p* = 0.57); WC: waist circumference (*p* = 0.69); SBP: systolic blood pressure (*p* = 0.365); DBP: diastolic blood pressure (*p* = 0.392); Glycemia (*p* = 0.349); Triglycerides (*p* = 0.601); C-HDL: high-density lipoprotein (*p* = 0.235)

Given the results, to elucidate clinically relevant and significant changes between the different allocation groups, we decided to perform a post-hoc analysis of the percentual balance of the risk factors (components). We evidenced a percentage difference of 27.4% -*p* > 0.05- (increasing 14.3% in CG and reducing 13.1% in IG) in metabolic syndrome development at 6 month of intervention. In this regard, looking at the different components of the metabolic syndrome in both groups, a significant percentage reduction was observed in the IG for waist circumference, abdominal circumference, triglycerides and HDL-C. Nevertheless, this percentage increased in the CG, giving an overall percentual balance of 13% (*p* = 0.69), 13.5% (*p* = 0.601) and 9.5% (*p* = 0.599), respectively. In addition, we found a significant diastolic blood pressure reduction in the IG (-21.8%, *p* = 0.392), and the systolic blood pressure remained without changes (*p* = 0.365). In the CG, there was a percentage increase. The glycaemic profile worsened (both groups' blood glucose levels were higher). However, this increase was lower in the IG -*p* < 0.05- (17.4% vs 38.4%). Figure S2 shows the components of MS evolution at baseline and six months of intervention in CG and IG. Likewise, Fig. 4 shows the percentual balance between the components of MS and lipid profile at six months of intervention in both groups. These results reached non-statistically significant trends, probably due to the limited sample size.

Finally, cardiovascular risk was analysed at baseline and after the intervention using the REGICOR and SCORE risk functions. According to the REGICOR equation, for the development of cardiovascular disease at ten years, we found a greater percentage reduction in the mean risk of 1.02% in the IG compared to the CG. Similarly, according to SCORE, a 0.12% lower percentual balance was obtained in IG (0.22%) compared to CG (0.34%) in terms of stratified risk.

## Discussion

The present study demonstrated that, after implementing a nutritional programme focused on high prebiotic and probiotic dietary modulation in patients diagnosed with schizophrenia spectrum disorder, the anthropometric profile (all variables) and, therefore, the risk of MS improved significantly in the IG. Similar results were obtained by Caemmerer et al. (2012) and Sugawara et al. (2018) [25, 26]. Likewise, the intervention reduced the risk of morbidity and mortality associated with cardiovascular events. Despite the limited resulting percentage, as noted by Dabke et al. (2019), the association of the nutritional programme with the conventional therapeutic approach led to a high synergistic impact on the improvement of dysmetabolic states [8], which are very important frequent in this population [13, 15, 16]. In this sense, the clinically significant results obtained support the meta-analysis developed by Teasdale et al. (2017), showing non-pharmacological interventions (dietary modulation and nutritional education) are established as coadjuvant therapies for metabolic anomalies [32], improving tolerance and pharmacological acceptance rates [24].

Regarding the level of compliance and results obtained in both allocation groups, it is essential to highlight the contextual framework of the global SARS-CoV-2 pandemic in which this clinical trial has been developed. In this regard, as indicated by Solé et al. (2021), most preliminary studies during the current pandemic have focused on psychological distress in the general population [9]. However, this study has been the first to address cardio-metabolic improvement through a nutritional intervention focused on prebiotics and probiotics dietary modulation in schizophrenia spectrum disorders. Nevertheless, numerous associated difficulties have probably prevented us from obtaining more statistically significant results. In this sense, the particular vulnerability of the target population in a context of confinement and global pandemic [9, 11, 12] stands out. This situation affects tangentially and negatively physical health [23, 32]. Thus, evidence shows a high complexity approach to schizophrenia [2], to which a context of confinement and social restriction has been added. Furthermore, there is proof of the effect of this situation on these patients, who see limited adherence to coping strategies, encouraging an increase in unhealthy lifestyles [9] related to inadequate dietary and physical exercise patterns [11, 32]. This fact may have modified the response to the symbiotic approach under investigation [8, 23].

Scientific evidence supports and clarifies the results obtained in the present study, with an increase in cardiovascular risk factors and associated morbidity and mortality during the SARS-CoV-2 era, especially in susceptible subjects and those with pre-existing MS [12, 32, 33]. This situation seems to be linked to a modification of lifestyles and defined by hypercaloric dietary patterns and restrictions in physical activity (up to 60% lower) [10, 12, 32]. These conditions can increase body weight and worsen the glycaemic and lipid profile, even in the short term [12, 23, 34]. In this sense, Solé et al. (2021) and Rishi et al. (2020) support the need to develop new strategies for home-based care and monitoring in states of confinement, promoting appropriate lifestyles and optimal health outcomes [9, 23]. Furthermore, these strategies should be strongly supported by technological development [11].

On the other hand, clinical trials with nutritional supplements or dietary approaches in the absence of psychopharmacological treatment are limited [8] and show marked heterogeneity and lack of methodological rigour [2, 5]. However, although the results obtained in the literature are not consistent, the findings of Patra (2016) support our findings, where the multimodal symbiotic approach, with nutraceutical action, was found to be effective as a complementary strategy in the treatment of dysmetabolic states in schizophrenic disorders [7].

Finally, and in agreement with Balanzá (2017), it is necessary to highlight the role of advanced practice nursing, a cornerstone in the multidisciplinary approach and the main responsible for the effectiveness of dietetic-nutritional interventions and improvement of lifestyles in the psychiatric population [17]. Thus, contrary to what was stated by Teasdale et al. (2017) [32], the proposed interventions' effectiveness is influenced by dieticians' participation and other health professionals with nutrition skills. In this group, nurses highlight because they can provide added value in the holistic care of the psychiatric patient [14] for several reasons: (i) assuming a prominent role in the coordination of caring for this population, (ii) their capability to offer care in different settings (highlighting home care) and (iii) optimal multimodal management of socio-health resources in states of social restriction and confinement [9, 17, 23].

### Limitations

The main limitations of the present study are related to the sample size and the possible loss or lack of cooperation of participants in the intervention phase. However, this limited sample size could explain the few significant differences in biochemical and cardiovascular profiles. Furthermore, regarding the associated cardio-metabolic diagnosis, it should be noted that a minority of the participants were on pharmacological treatment prior to the study.

It is worth highlighting the exclusion of those subjects with a potential risk of cardiovascular health problems during the debut or exacerbation of the underlying psychopathological process. This decision was taken because the research focused on chronic patient management. Consequently, the results cannot be extrapolated to a population in the initial stages of exacerbation of the disease.

In addition, this work does not show the changes in the dietary pattern of the subjects during the intervention, which may limit the significance and clinical relevance of the results obtained. Their analysis in further research would help clarify which nutritional factors are closely linked to improving the cardio-metabolic profile in the target population.

On the other hand, the available evidence on the subject of the study makes it difficult to contrast the results obtained in different health care settings.

Finally, it is essential to note that this study was conducted during the SARS-CoV-2 pandemic, making it difficult to achieve the proposed intervention. Furthermore, it is necessary to consider the inherent characteristics of the subjects under study, a population particularly vulnerable to change, especially in a context of confinement and a global pandemic.

## Conclusions

The development of a dietary-nutritional intervention with high symbiotic content in patients diagnosed with schizophrenia spectrum disorders has been postulated as an effective and clinically significant therapy in reducing cardiovascular risk factors and improving metabolic outcomes in the context of the global SARS-CoV-2 pandemic. These dietary recommendations may work as an adjuvant in the metabolic syndrome of patients with schizophrenia spectrum disorders, leading to increased pharmacological tolerance and improved physical health. Thus, a decrease of 14.3 percentage points in the prevalence of MS in the IG has been observed, compared to an increase of 13.1 points in the CG, which represents a differential balance of 27.4% between groups. Furthermore, despite the target population's inherent lifestyle dysfunctionalities, prebiotics and probiotics have been shown to offer a relevant and promising solution in different application contexts, improving patients' quality of life and mitigating the risk of associated cardio-metabolic disorders. Nursing plays a prominent role in achieving optimal health outcomes, being a cornerstone in the multimodal approach and modulating lifestyles, through dietary-nutritional education. Finally, further studies with larger sample sizes are needed to corroborate these promising results.

## Supplementary Information

**Additional file 1: Table S1.** Anthropometric Assessment and Physical HealthRecord. **Table S2.** WeeklySymbiotic Diet Register. **Figure S1.** Nutritional Information. **Figure S2.** Evolution of components of Metabolic Syndrome atbaseline and six months of intervention: Control group and intervention group.

## Data Availability

The collected data that support the findings of this study are available on reasonable request from the corresponding author.
