# Supplementary material for: Impact of high prebiotic and probiotic dietary education in the SARS-CoV-2 era: improved cardio-metabolic profile in schizophrenia spectrum disorders
Source: BMC Psychiatry. 2022 Dec 12;22:781. doi: 10.1186/s12888-022-04426-9 (PMC9743108; doi:10.1186/s12888-022-04426-9)
Supplement: Supplementary file 1 — Additional file 1: Table S1. Anthropometric Assessment and Physical HealthRecord. Table S2. WeeklySymbiotic Diet Register. Figure S1. Nutritional Information. Figure S2. Evolution of components of Metabolic Syndrome atbaseline and six months of intervention: Control group and intervention group. [file 12888_2022_4426_MOESM1_ESM.docx]

**SUPPLEMENTARY MATERIAL**

**Table S1. Anthropometric Assessment and Physical Health Record.**

| **[ BASAL ]** | | | **-**  **MONTH No. 1-** | | | **-**  **MONTH No. 2-** | | | **-**  **MONTH No. 3-** | | |
| --- | --- | --- | --- | --- | --- | --- | --- | --- | --- | --- | --- |
| **ANTHROPOMETRIC ASSESSMENT** | | | | | | | | | | | |
| **-**  **WEIGHT** (kg): | **-**  **ABDOMINAL GIRTH** *(cm):* | | **- WEIGHT** (kg): | **- ABDOMINAL GIRTH** *(cm):* | | **- WEIGHT** (kg): | **- ABDOMINAL GIRTH** *(cm):* | | **- WEIGHT** (kg): | **- ABDOMINAL GIRTH** *(cm):* | |
| **-**  **SIZE** *(cm):* | **-**  **BMI** *(weight/height^2^)* | | **- SIZE** *(cm):* | **- BMI** *(weight/height^2^)* | | **- SIZE** *(cm):* | **- BMI** *(weight/height^2^)* | | **- SIZE** *(cm):* | **- BMI** *(weight/height^2^)* | |
| **CARDIOVASCULAR CONTROL** | | | | | | | | | | | |
| **- BLOOD PRESSURE** *(mmHg):* | | **-**  **HEART RATE** *(ppm):* | **- BLOOD PRESSURE** *(mmHg):* | | **- HEART RATE** *(ppm):* | **- BLOOD PRESSURE** *(mmHg):* | | **- HEART RATE** *(ppm):* | **- BLOOD PRESSURE** *(mmHg):* | | **- HEART RATE** *(ppm):* |

- **OBSERVATIONS:**

| **-**  **MONTH No. 4 -** | | | **-**  **MONTH No. 5 -** | | | **-**  **MONTH No. 6 -** | | |
| --- | --- | --- | --- | --- | --- | --- | --- | --- |
| **ANTHROPOMETRIC ASSESSMENT** | | | | | | | | |
| **- WEIGHT** (kg): | **- ABDOMINAL GIRTH** *(cm):* | | **- WEIGHT** (kg): | **- ABDOMINAL GIRTH** *(cm):* | | **- WEIGHT** (kg): | **- ABDOMINAL GIRTH** *(cm):* | |
| **- SIZE** *(cm):* | **- BMI** *(weight/height^2^)* | | **- SIZE** *(cm):* | **- BMI** *(weight/height^2^)* | | **- SIZE** *(cm):* | **- BMI** *(weight/height^2^)* | |
| **CARDIOVASCULAR CONTROL** | | | | | | | | |
| **-**  **BLOOD PRESSURE** *(mmHg):* | | **- HEART RATE** *(ppm):* | **- BLOOD PRESSURE** *(mmHg):* | | **- HEART RATE** *(ppm):* | **- BLOOD PRESSURE** *(mmHg):* | | **- HEART RATE** *(ppm):* |

- **OBSERVATIONS:**

**I'VE EATEN THIS WEEK**

**Table S2. Weekly Symbiotic Diet Register.**


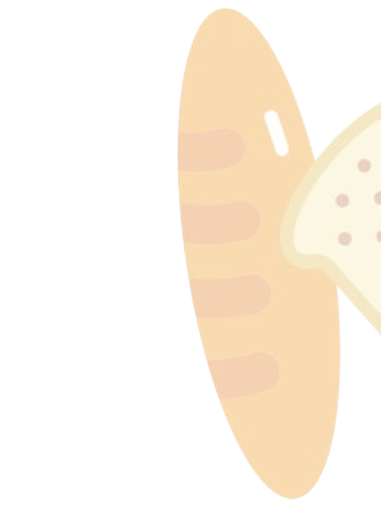

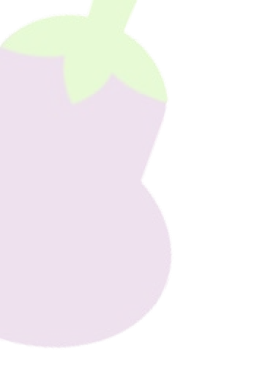

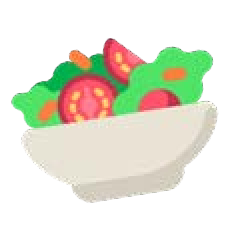

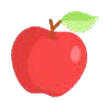

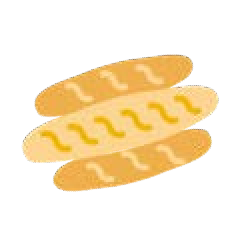

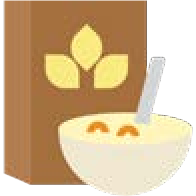

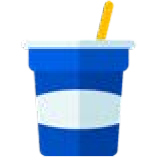

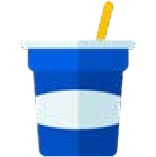

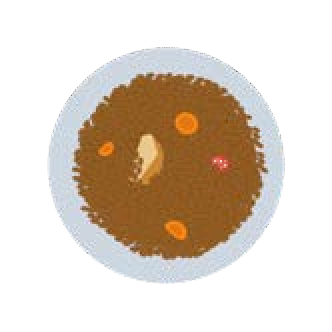

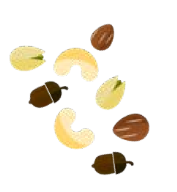

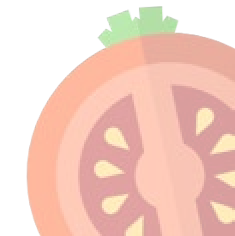


**Mark with an x the options you have included in your daily menu.**


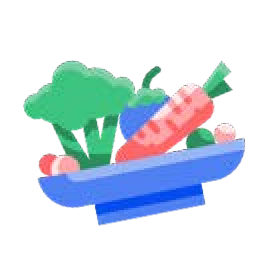
**WEEK ____________**


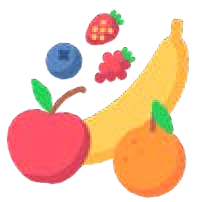

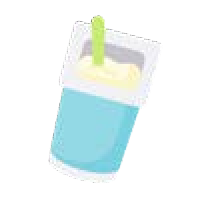

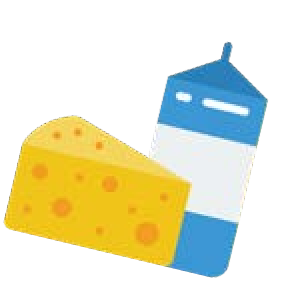

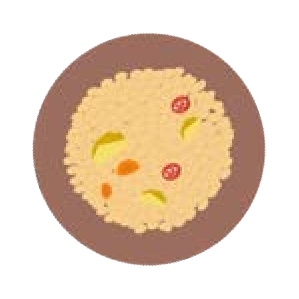


|  | **M** | **T** | **W** | **T** | **F** | **S** | **S** | **TOTAL**  **DAYS** |
| --- | --- | --- | --- | --- | --- | --- | --- | --- |
| **1 salad plate with vegetables of different colours.** |  |  |  |  |  |  |  |  |
| **1 plate of cooked vegetables (steamed, boiled, sautéed, baked, etc.).** |  |  |  |  |  |  |  |  |
| **At least one piece of fruit.** |  |  |  |  |  |  |  |  |
| **More than one piece of fruit.** |  |  |  |  |  |  |  |  |
| **Cereals such as oats or rye, either as whole grain bread, in homemade pastries or**  **mixed with milk or yogurt.** |  |  |  |  |  |  |  |  |
| **At least one natural yogurt.** |  |  |  |  |  |  |  |  |
| **More than one natural yogurt.** |  |  |  |  |  |  |  |  |
| **Other types of fermented dairy such as cottage cheese, fresh cheese, milks**  **fermented or kefir.** |  |  |  |  |  |  |  |  |
| **A plate of vegetables (lentils, beans, chickpeas, broad beans or peas).** |  |  |  |  |  |  |  |  |
| **A stew prepared with onion and tomato sauce (it can be**  **vegetables).** |  |  |  |  |  |  |  |  |
| **A handful of raw nuts.** |  |  |  |  |  |  |  |  |

**Figure S1. Nutritional Information**

**THE STAR FOODS**

*These foods contain the largest amount of fiber fermentable by the bacteria in our colon. Try to include at least 1 each day.*

##
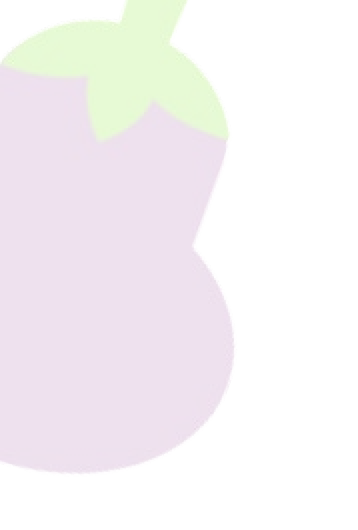

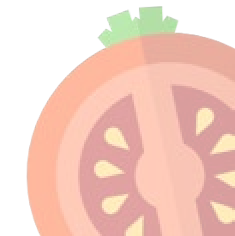
VEGETABLES:


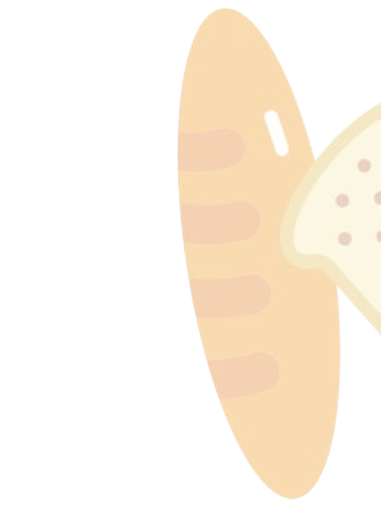

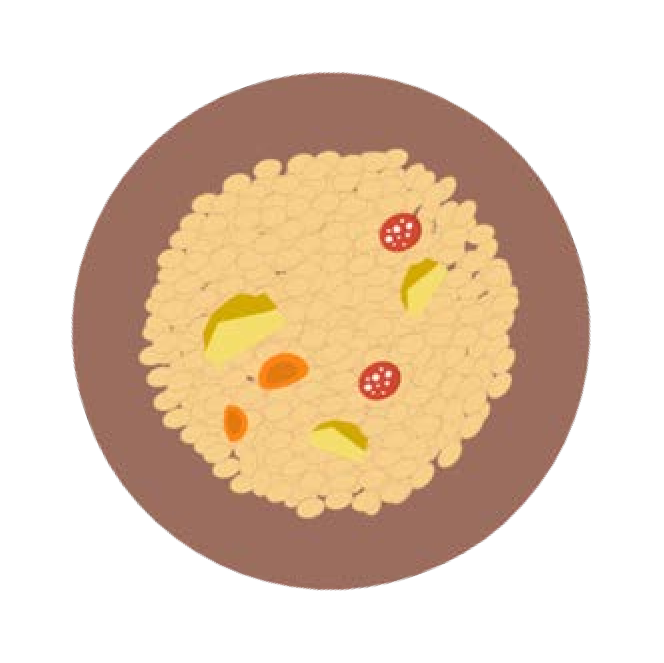

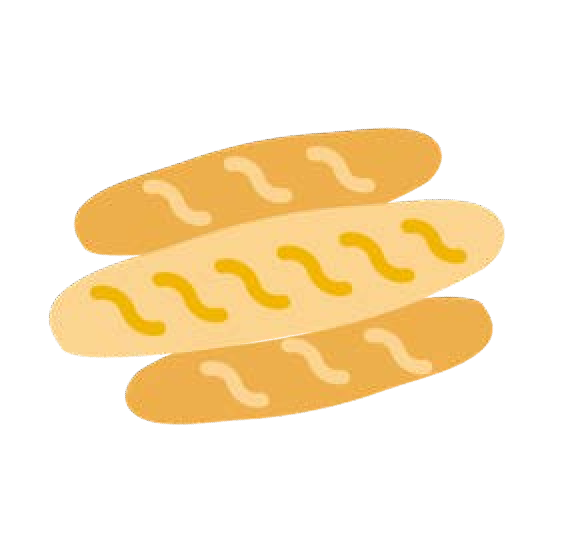

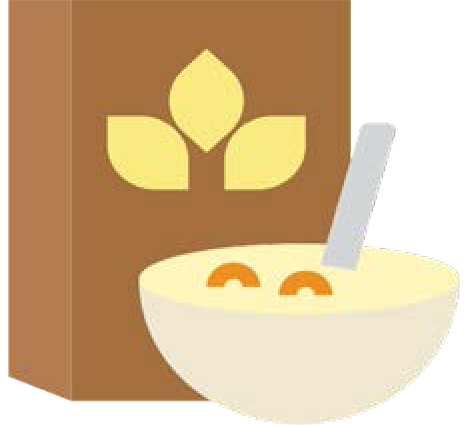


-
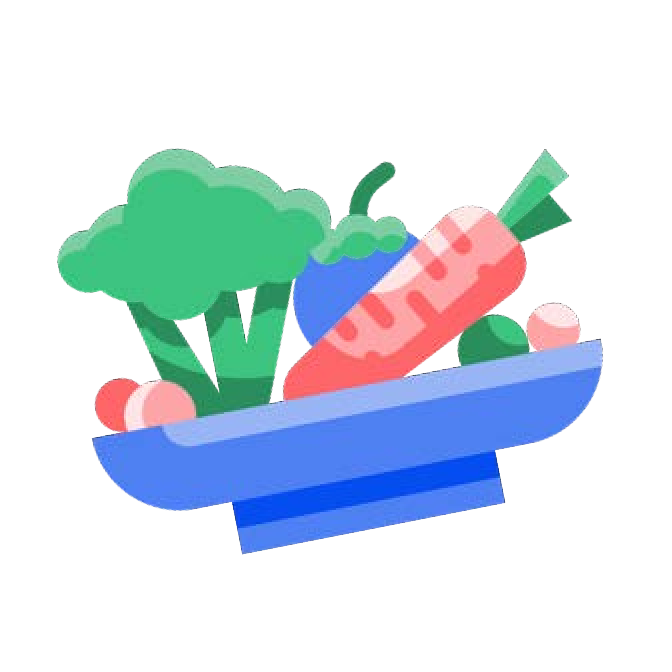
Onion
- Asparagus
- Artichokes
- Leek
- Tomato
- Cucumber
- Carrot


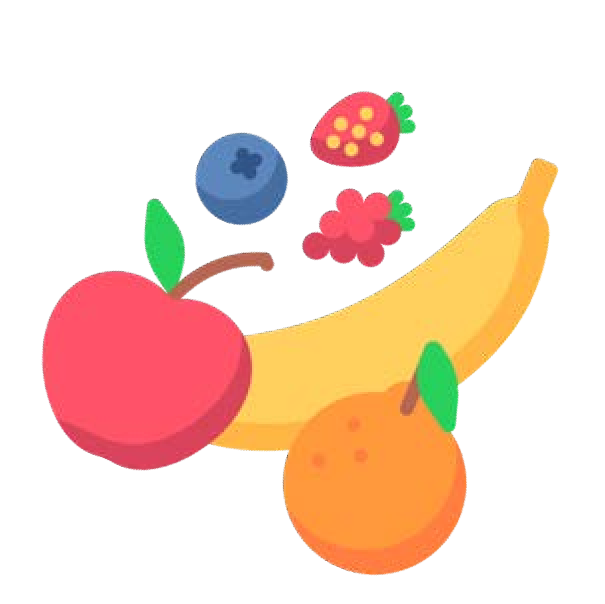
***FRUITS:***

Banana; Apple (better with skin);

Orange; Tangerine; Blueberries; Grapes

***LEGUMES:***

- - Beans
  - Peas

***CEREALS:***

- Oats
- Rye

**HEALTHY EATING RECOMMENDATIONS**

Try to always eat your whole grain. Although they provide us with the same amount of energy, they contain a greater amount of fiber and nutrients that are very necessary for the maintenance of health.

You must eat at least 2 portions of vegetables a day. One of them should be in the form of raw vegetables, for example, in salad. Remember to include vegetables of different colours.


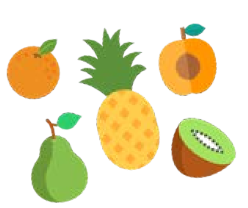

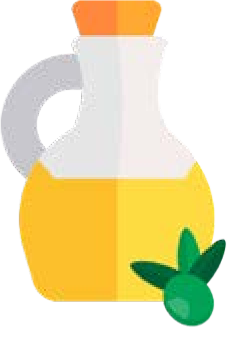

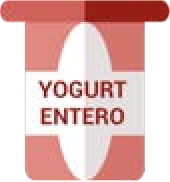

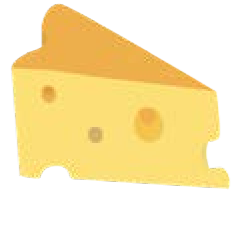

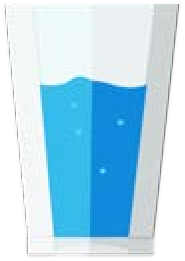


Includes vegetables 2 to 3 times a week. These can be eaten in stews, in stir-fries with vegetables, or in salads.

You can also eat them as a garnish for meat, fish and eggs.

Eat 2 to 3 pieces of fresh fruit every day.

You should consume between 3 and 4 portions of fish per week, alternating between white (hake, cod, monkfish, sole, etc.) and blue (anchovy, sardine, salmon, emperor, tuna).

Remember to include 2 to 3 portions of dairy every day, and try to have at least one portion of fermented dairy such as yogurt, fresh cheese or kefir.

Try to cook and dress your salads always with extra virgin olive oil.


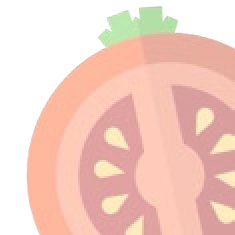


Always choose water as a drink at your meals.


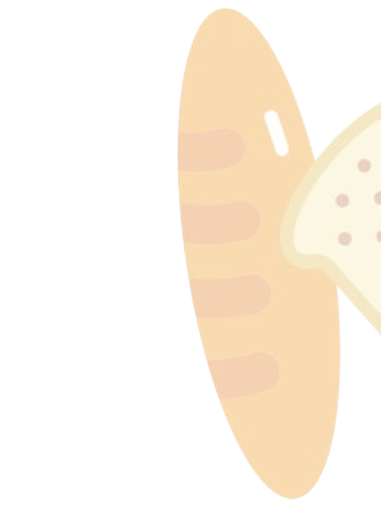

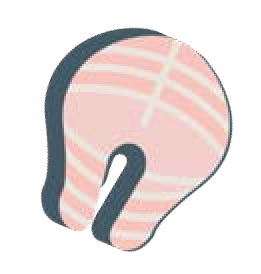

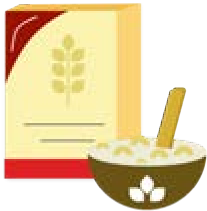

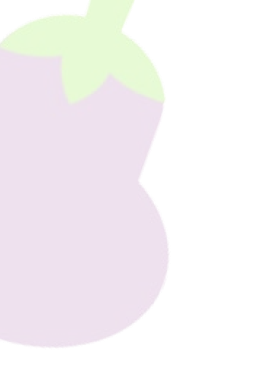

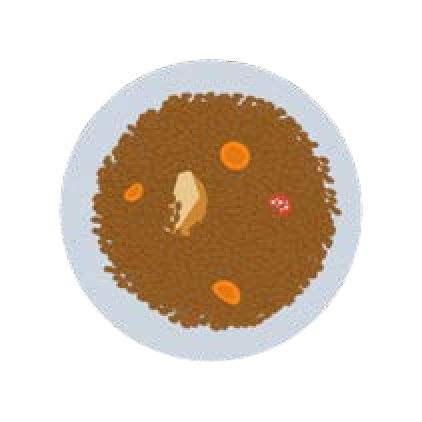

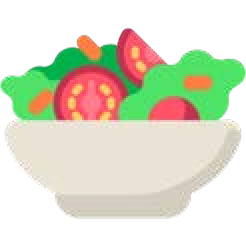


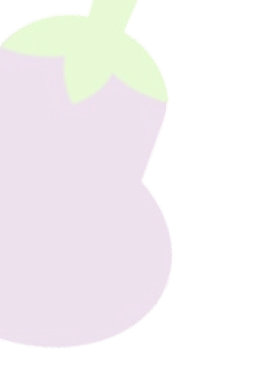

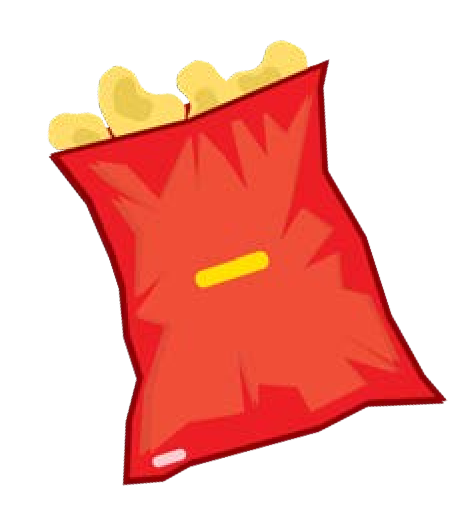

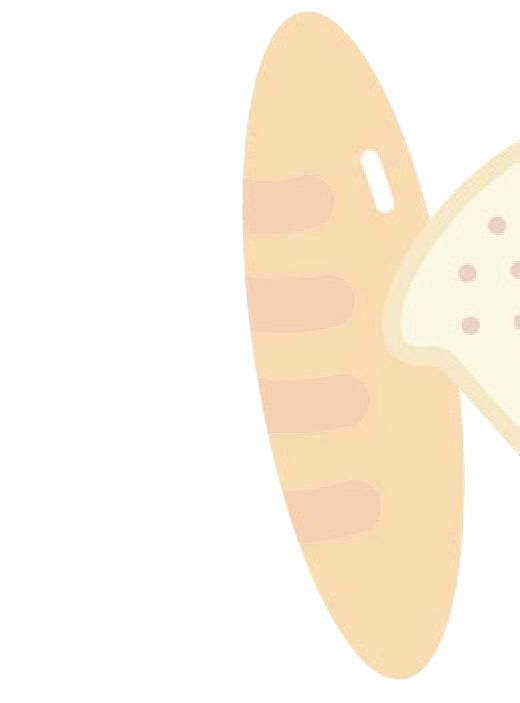
**HEALTHY EATING RECOMMENDATIONS**

**Highly processed foods such as ready meals, sauces, snacks and pastries**

**AVOID:**

.


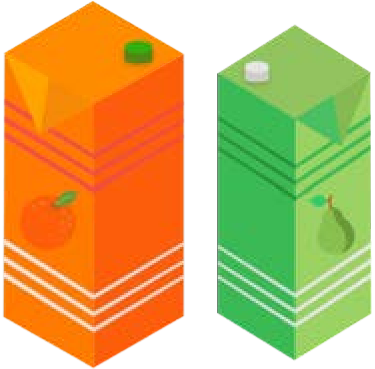


**The juices, even if they are natural.**

**They provide too much sugar**


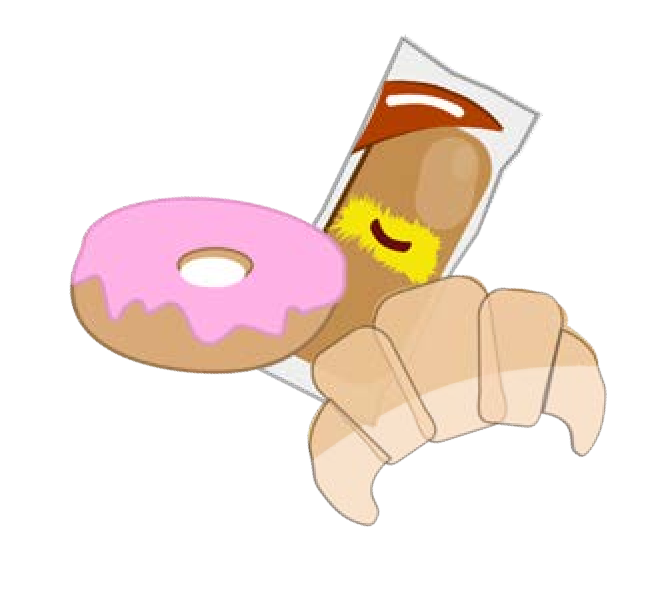


T

**Sugar, pastries and sweet desserts, especially if they are industrial. If you eat them, make sure they are only eaten on special occasions and prepared at home to ensure that quality ingredients have been used.**


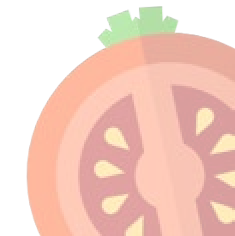

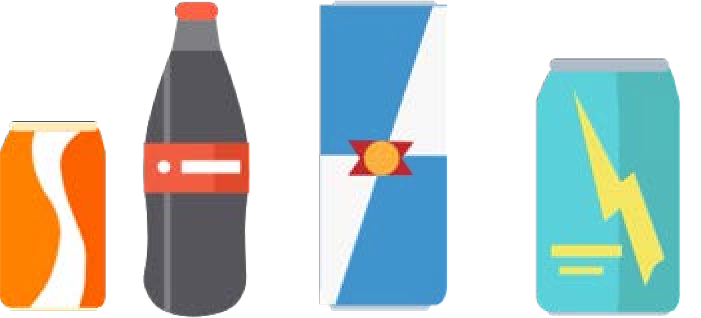


**Sugared soft drink.**


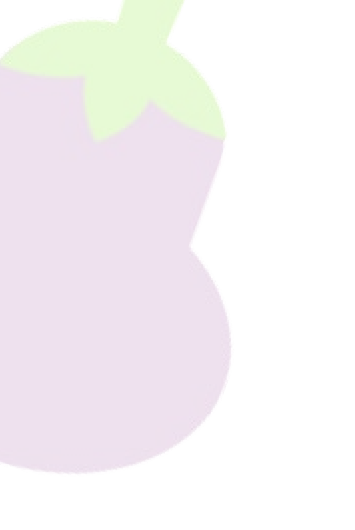
**HEALTHY EATING RECOMMENDATIONS**

|  | **MONDAY** | **TUESDAY** | **WEDNESDAY** | **THURSDAY** | **FRIDAY** | **SATURDAY** | **SUNDAY** |
| --- | --- | --- | --- | --- | --- | --- | --- |
| **BREAKFAST** | Coffee Yogurt with oats and blueberries | Tea Toasted flour  wholemeal rye with oil, tomato and ham | Oatmeal coffee with milk, banana, almonds and pure cocoa | Wholemeal Tea with Avocado and slices of  tomato | Coffee  Wholemeal rye toast with butter and jam without  sugar | Tea  Yogurt with oats and grapes | Coffee Homemade carrot cake with wholemeal |
| **MID-MORNING** | Banana | Apple | Ham rolls cooked with rocket and cottage cheese | Cottage cheese with strawberries | Turkey rolls  with guacamole and tomato | Baked apple with cinnamon | Handful of almonds |
| **LUNCH** | Salad with tuna and avocado Stewed beans with vegetables Orange | Skipped from  leeks and asparagus with prawns  Baked chicken leg with baked potato  Pear | Chickpea hummus with carrot sticks  Grilled salmon with spinach  2 Tangerines | Potato omelette with sautéed peppers Apple | Sauteed peas with onions  Chicken wings with  Banana wine | Salad with cured cheese Stewed potatoes with chicken Infusion | Lettuce hearts with anchovies Paella Orange |
| **SNACK** | 1 handful of nuts | 1 handful of cashew nuts | Infusion | A handful of toasted almonds | Natural yogurt with cashew nuts | Banana | Coffee with milk |
| **DINNER** | Cream of  zucchini with grated parmesan cheese  Grilled Hake Natural Yogurt  with cinnamon | Salad of cherry tomatoes, fresh cheese and black olives  French omelette | Sauteed mushrooms with garlic  Grilled turkey  Natural yogurt | Spinach and goat cheese salad Sole Natural yogurt | Salad with hard-boiled egg and mackerel Orange | Sautéed artichokes with garlic and paprika French omelette  Natural yogurt | Sautéed spinach with pine nuts Grilled pork fillet  Natural yogurt |


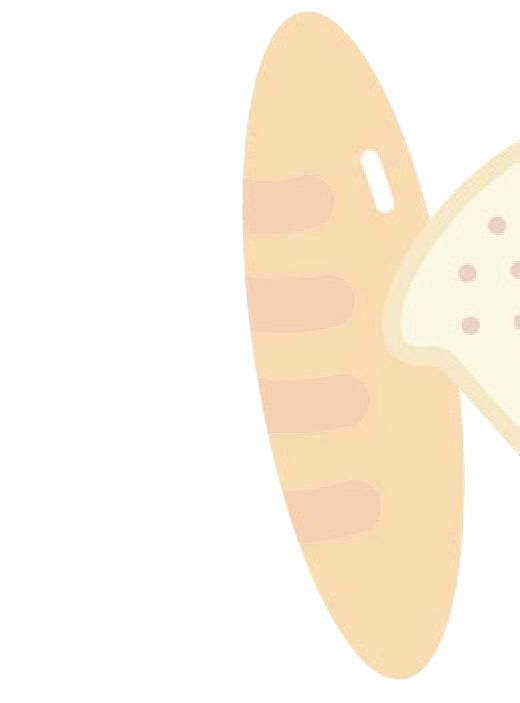

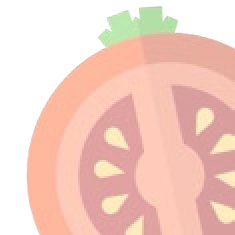


**Figure S2. Evolution of components of Metabolic Syndrome at baseline and six months of intervention: Control group and intervention group.**


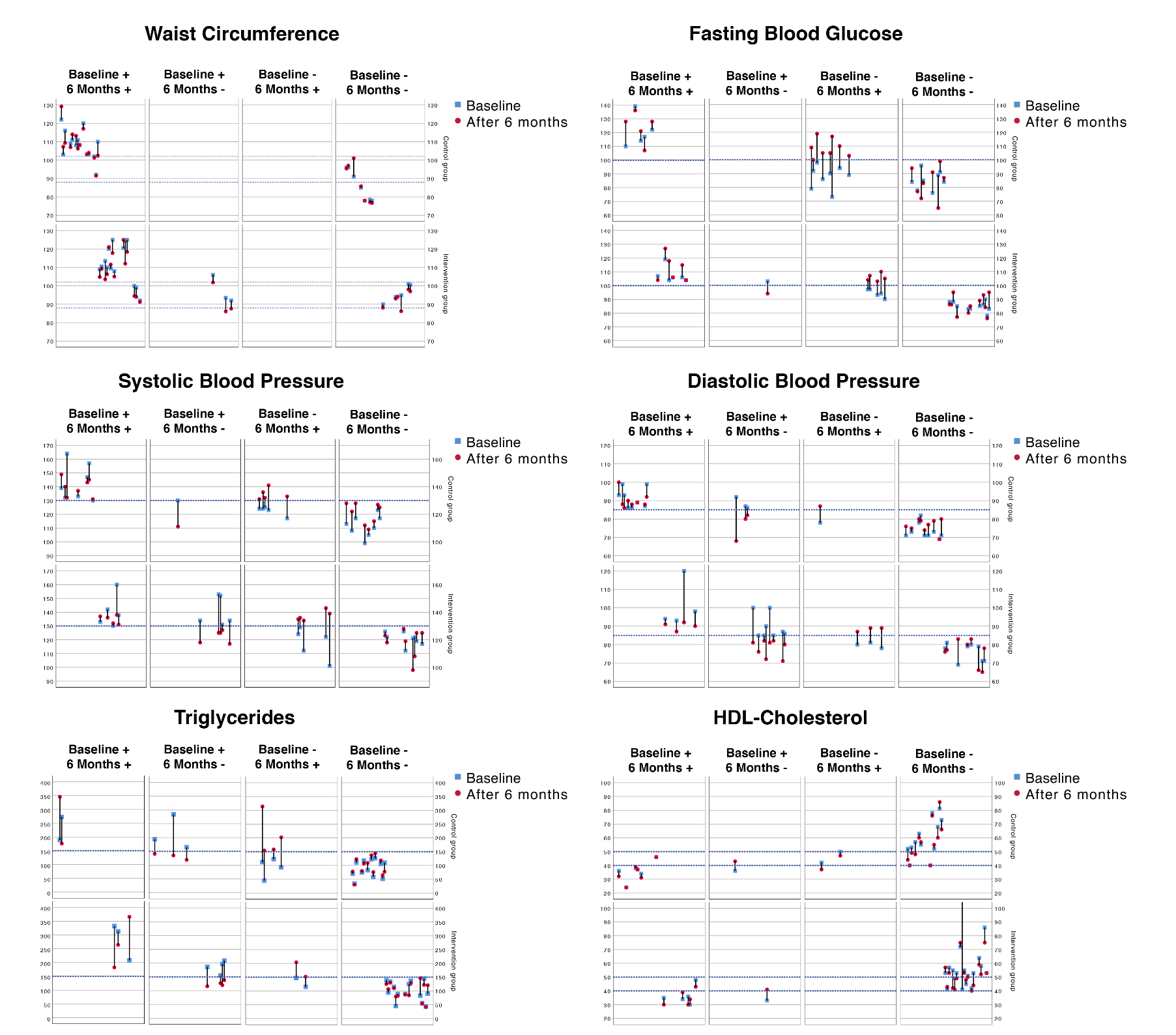


*Waist Circumference (cm); Fasting Blood Glucose (mg/dL); Systolic Blood Pressure (mmHg); Diastolic Blood Pressure (mmHg); Triglycerides (mg/dL); HDL-Cholesterol (high-density lipoprotein, mg/dL).* ***Baseline +/After 6 months +:*** *Evolution of MetS components between control and intervention group that have remained above the normal cut-off point (presence of MetS component), at baseline and after 6 months of intervention.* ***Baseline +/After 6 months -:*** *Evolution of MetS components between control and intervention group that were above the normal cut-off point (presence of MetS component), but which has been reduced below the cut-off point after 6 months of intervention (absence of MetS component).* ***Baseline -/After 6 months +:*** *Evolution of MetS components between control and intervention group that were bellow the normal cut-off point (absence of MetS component), but which has been increased after 6 months of intervention (presence of MetS component).* ***Baseline-/After 6 months -:*** *Evolution of MetS components between control and intervention group that have remained bellow the normal cut-off point (absence of MetS component), at baseline and after 6 months of intervention.*
